# Epsom Salts Made Palatable

**Published:** 1909-08

**Authors:** 


Epsom Salts Made Palatable.—A very fine preparation of epsom salts, according to Burnett Physicians Drug News, October, 1908, is made by taking two pounds of the salts, one ounce of the tincture of cardamon, twenty grains of vanillin, three drachms of saccharin, two ounces of alcohol, two ounces of glycerine, two ounces of coffee (roasted and ground) and water to make a half gallon. Stir the coffee in the half gallon of hot water and let it stand for fifteen minutes and let it draw, then add the salts, vanillin and tincture of cardamon to the alcohol and shake; then add the glycerine and the saccharin, and when the coffee mixture is cold enough, mix all together and filter. A half ounce of the salts is contained in each ounce of the mixture. It keeps well, acts well, tastes well, and children cry for it.—*Medical Fortnightly.*

